# Molecular detection of trophic interactions: emerging trends, distinct advantages, significant considerations and conservation applications

**DOI:** 10.1111/eva.12225

**Published:** 2014-10-29

**Authors:** Elizabeth L Clare

**Affiliations:** School of Biological and Chemical Sciences, Queen Mary University of LondonLondon, UK

**Keywords:** conservation biology, ecological genetics, metabarcoding, molecular dietary analysis, species interactions

## Abstract

The emerging field of ecological genomics contains several broad research areas. Comparative genomic and conservation genetic analyses are providing great insight into adaptive processes, species bottlenecks, population dynamics and areas of conservation priority. Now the same technological advances in high-throughput sequencing, coupled with taxonomically broad sequence repositories, are providing greater resolution and fundamentally new insights into functional ecology. In particular, we now have the capacity in some systems to rapidly identify thousands of species-level interactions using non-invasive methods based on the detection of trace DNA. This represents a powerful tool for conservation biology, for example allowing the identification of species with particularly inflexible niches and the investigation of food-webs or interaction networks with unusual or vulnerable dynamics. As they develop, these analyses will no doubt provide significant advances in the field of restoration ecology and the identification of appropriate locations for species reintroduction, as well as highlighting species at ecological risk. Here, I describe emerging patterns that have come from the various initial model systems, the advantages and limitations of the technique and key areas where these methods may significantly advance our empirical and applied conservation practices.

**Definition of terms**

*Amplicon* refers to the region of DNA that has been amplified by targeted primers for sequencing.

*Connectance* in food webs describes the degree to which trophic levels are associated.

*DNA barcoding* in the current global sense refers to an international programme to assemble a reference library for biological diversity based on a single target sequence for animals, the cytochrome *c* oxidaze subunit 1.

*eDNA* refers to trace material left behind in the environment, e.g. from hairs shed or cells left in faeces.

*Generality* in food webs describes the average number of species at the lower level using the higher level.

*High-throughput sequencing* or next generation sequencing (NGS) is a process where many millions of sequences are generated simultaneously, often from mixed slurries of material.

*Linkage density* in food webs describes the average number of interactions made by species within the networks.

*Metabarcoding* (often considered a branch of metagenomics) is the process by which we sequence millions of copies of a specific target region of the genome from a mixed slurry of material. Unlike genomics where we recover every gene in one genome, metagenomics recovers one gene in many genomes.

*Metagenomics* (often referred to as a broad category which includes metabarcoding) may refer to the application of genomic techniques to assessments of diversity in the general sense but more specifically refers to the assembly of entire genomes from a diversity of species within a mixed sample to differentiate it from metabarcoding (above).

*MID tags* are small nucleotide sequences built into primers of generally 10 bp or less. Each PCR can be assigned a different MID which can then be used to separate samples after sequencing. These are occasionally called libraries or barcodes though the latter creates confusion when used with ‘DNA barcoding’ and ‘metabarcoding’.

*Sanger sequencing* is the traditional process of producing a single DNA sequence for every extracted sample and PCR reaction.

*Vulnerability* in food webs is the average number of species at the higher level using the lower level.

## Introduction

**Figure 1:**
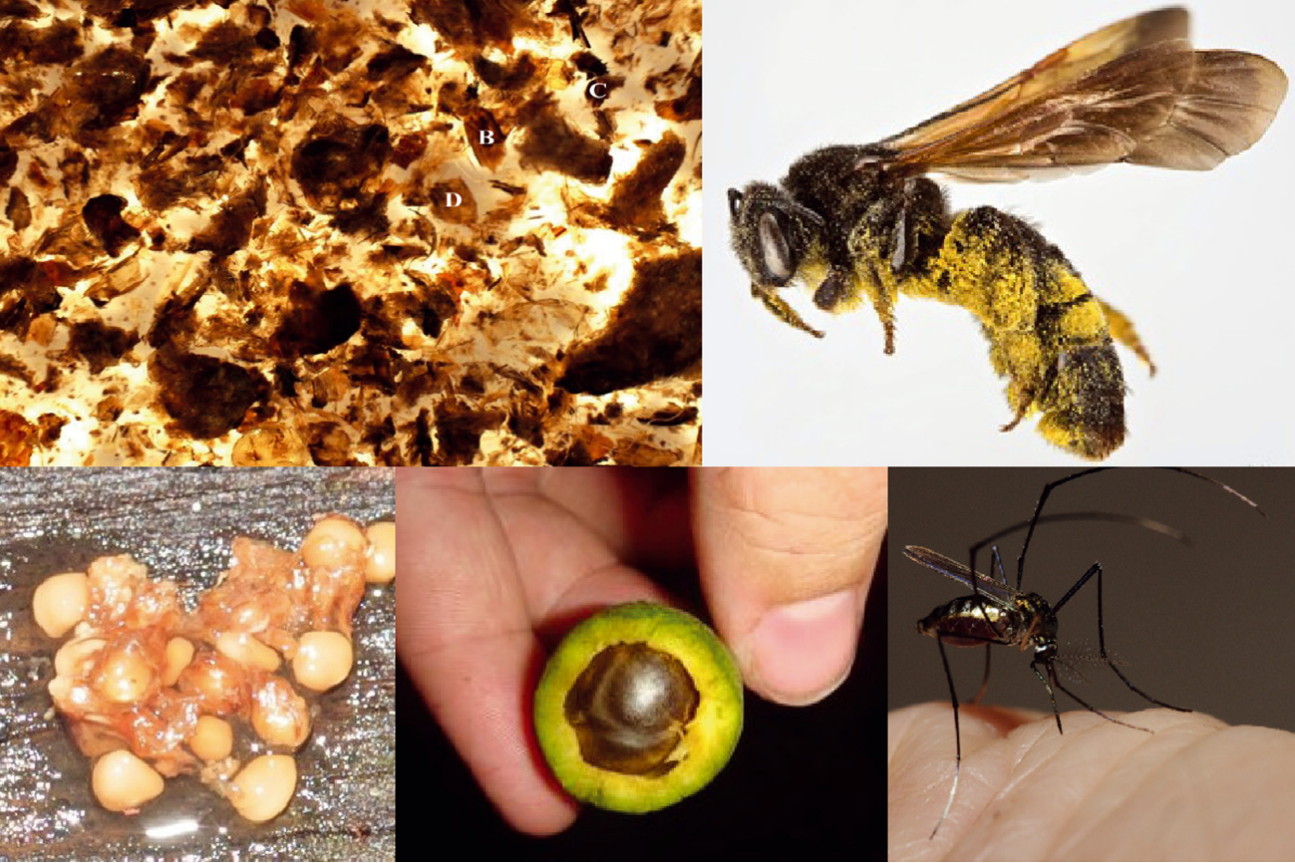
A wide variety of interactions occur in nature and all cases leave behind traces of environmental DNA. Clockwise starting at top left, DNA from crushed insects (B, C, D) in faeces can identify the insect prey and the predators DNA is present in traces, bees carry pollen, which provides plant DNA, parasites blood meals are a source of DNA from visited animals, chewed seeds have saliva and deposited seeds epithelial cells, which can be used to identify the dispersing animal (Photographs used with permission: mosquito – M. Brock Fenton, bee – L. Packer and Bee Tribes of the World, all others E.L. Clare).

Species’ interactions are the basis of ecosystem functioning and the provision of ecosystem services (Keesing et al. 2010; Kunz et al. 2011). Such interactions underlie evolutionary and ecological principles and may be competitive (e.g. predators and prey, parasites and hosts, individuals for resources) or mutualistic (e.g. pollen and seeds for dispersers; Fig.1). These relationships are the building blocks of interaction networks (e.g. food webs), and understanding their structural mechanisms is crucial to predicting their response to disturbance. Despite their importance, it is much easier to count species in an ecosystem than to characterize their interactions (McCann 2007) and the limitations of direct observation mean that quantifying relationships and their structural mechanisms remains challenging. Despite this, an accurate account of how species interact within their environment is fundamental to the establishment of good conservation practice both in a theoretical context, for example understanding how and why species may persist or be threatened, and also in applied practice, for example managing reintroductions and long-term monitoring.

Historically, accurate quantification of interactions in a community has been difficult or impossible because of the number of potentially interacting species, particularly when generalists or omnivores are common and resources diverse such as in tropical environments. The development of molecular methodologies and, in particular, high-throughput sequencing (HTS) techniques now provide a robust means of accurately and cost-effectively examining biodiversity at a scale and level of precision not previously available. When applied to species interactions, these methods deliver an unprecedented level of insight into ecological networks, making it possible to simultaneously assess thousands of interactions and providing a powerful tool for conservation biology. This approach will be particularly effective if measured over time and space allowing us to better predict functional responses to environmental change.

**Figure 2:**
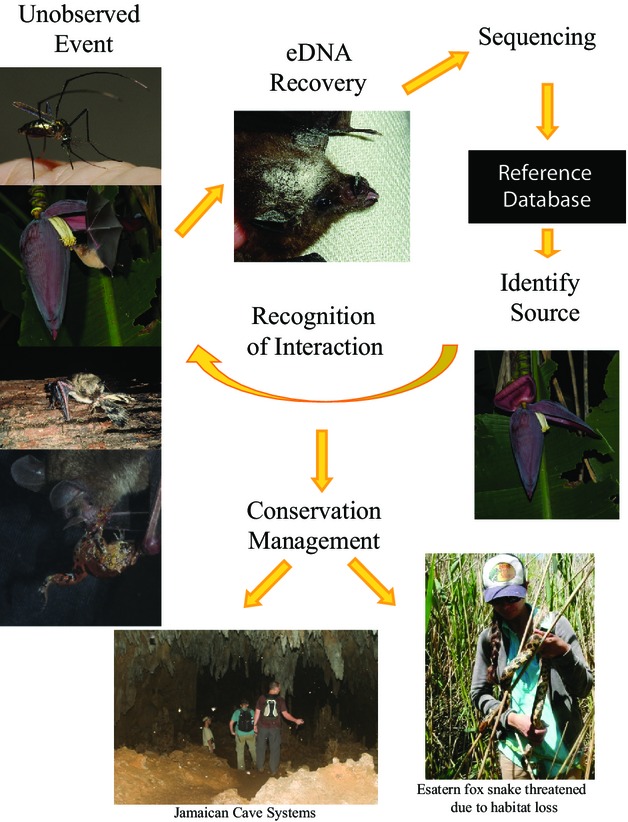
The analytical chain for molecular analysis. High-throughput sequencing platforms coupled with the public databases of sequences from a wide variety of taxa allow us to document species interactions. An unobserved event can be identified by sequencing eDNA (e.g. from pollen on a bat). The resulting unknown sequence can be compared against collections of taxonomically validated references for species-level documentation of the ecological event. This enables large-scale measurements of species’ interactions to be partly automated. The resulting databases can be used to quantitatively measure a variety of ecologically and evolutionarily important events, such as the relative niche flexibility of taxa, competition between taxa or the response of an ecological system to disruption. For example, resource use by bats in Jamaican cave systems have been a particular target of molecular studies (Emrich et al. 2014). Photographs used with permission: mosquito – M. Brock Fenton, bat with pollen – J. Nagel, fox snake – C. Davy all others E.L. Clare.

Molecular tools provide the potential for rapid species-level resolution of interactions. They do this by accessing DNA traces left behind (so called environmental DNA or eDNA) such as saliva on a chewed fruit or gut epithelial cells on deposited seeds, prey DNA contained in predator scats, or pollen carried by a bee, moth or bat (Fig.1). All of these traces may potentially be used to recreate the unobserved interaction event by sequencing target DNA which is unknown and matching it to a database of known sequences to identify its taxonomic origin (Fig.2). While conceptually simple, the technique is complex and vulnerable to methodological problems but, as I shall outline below, it is also providing fundamentally new insights into ecosystem dynamics.

A general trend towards the use of eDNA for ecological and evolutionary applications is apparent; for example, traces of DNA may be used to identify and study population dynamics (Taberlet and Fumagalli 1996), for the detection of invasive species (Dejean et al. 2012) or for biomonitoring of species at risk (Thomsen et al. 2012). However, the direct analysis of interactions between species through eDNA has been developing rapidly over the last 5–10 years, particularly since next-generation sequencing technologies became widely available. There are two generalized approaches to these assessments. Metagenomics relies on the amplification of all DNA in a sample and the recovery of all or part of the genome for any taxa present. This can be thought of as the ‘information-heavy’ approach where maximal taxonomic data are recovered on common species in the sample, but many rare taxa may be missed. The opposite approach is metabarcoding where the goal is to maximize taxonomic coverage by assessing only one or a few genes per species but in a comparatively broad way where rare taxa are likely to be detected. Metagenomics provides the opportunity to ask questions such as ‘what is the diversity of metabolic genes from this sample in an extreme environment’ while metabarcoding might address ‘what is the total diversity of this particular sample and is it higher or lower than one from elsewhere’. Given a finite sequencing effort, there is a clear trade-off between maximizing information per taxon versus maximizing taxonomic recovery itself (Srivathsan et al. 2014) and the appropriateness of a method will depend largely on the question and study system. Within both approaches, there are applications to environmental assessment (e.g. Bohmann et al. 2014) and specific diagnostics of trophic interactions (Symondson and Harwood 2014). These have different methodological approaches and analytical considerations. While this review is chiefly concerned with specific applications of metabarcoding to trophic interactions, where appropriate I will address these differences.

A growing number of papers in the last few years have introduced us to dietary analyses for insectivores, marine mammals, invertebrate predators and many more (reviewed in Symondson 2002; Pompanon et al. 2012), and while mutualistic interactions have proven more difficult to assess (Wilson et al. 2010; Clare et al. 2013b), herbivore networks are starting to appear (e.g. Newmaster et al. 2013). This is an exciting field and each new paper provides interesting conclusions which are changing how we view ecosystem functioning. While the technique is promising, it is not perfect and most authors must attempt to optimize their procedures and then acknowledge their limitations.

A number of excellent reviews in the last few years have summarized the history of molecular dietary analysis (Symondson 2002), best practices for the research approach (King et al. 2008), comparisons of approaches (Razgour et al. 2011) and a comprehensive overview of the promises of genomic techniques in molecular ecology (Pompanon et al. 2012). Given these resources, I will not attempt to recreate their work here, but I will consider two emerging trends – one from the world of parasites and one from the world of large vertebrates – that have been made possible by the application of molecular technologies. I will also examine a set of challenges that need to be considered, met and overcome in this emerging field before it can be effectively applied to answering conservation questions and in species conservation and management.

## Do we learn more from DNA?

While molecular analysis is becoming common within dietary studies because of its significant taxonomic resolution, there are key advantages of including traditional morphological analysis. For example, only morphology can efficiently allow us to distinguish different life stages of prey groups, for example the apparent consumption of adult versus pupal forms of Chironomidae, which represent subtle niche differentiation in trawling *Myotis* bats (Krüger et al. 2014). Thus, while molecular approaches may provide additional taxonomic resolution, they are not a universal improvement and there may be clear advantages of pairing multiple analytical techniques. But do we learn anything truly novel from molecular analyses or are we simply observing old trends with new data?

### Emerging patterns: how flexible are species?

Flexibility is one important component of ecosystem stability. Species with the capacity to adapt to environmental change are more resilient to habitat disruption. One of the most fascinating contrasts to emerge from species-level resolution afforded by molecular methods is the difference in flexibility between parasites and larger vertebrates. This key difference may have significant implications for species conservation.

### Increased specialization of parasites

Tachinid flies deposit their larvae in other insects and these larvae then consume their hosts. In two different studies conducted in Guanacaste, Costa Rica (Smith et al. 2006, 2007), Sanger sequencing methods were used to examine host specificity of tachinid parasitism. Within the genus *Belvosia*, morphological analysis suggested 20 distinct species, three of which were categorized as taxonomic generalists; however, the application of molecular methods suggested these actually represented 15 cryptic taxonomic specialists (Smith et al. 2006). The distributions of the hosts correspond to distinct wet and dry environments and appear to limit some of the parasites’ distributions. As a net result, 20 morphospecies are actually now thought to be 32 distinct lineages, and the degree of niche specialization is much higher than previously suggested but dictated by a complex interaction between host and environment (Smith et al. 2006). The same pattern was observed in a wider sample of tachinids when these authors specifically targeted a series of 16 presumed generalists and uncovered an unexpected 73 species (Smith et al. 2007). Of these original 16 morphospecies, some were true generalists, others represented a pair of cryptic species both of which were generalists, others a complex of multiple species including a generalist and many specialists but most represented a complex containing all unrecognized specialists (Smith et al. 2007). The trend towards increased specificity appears to be upheld in diverse environments. For example, in North America, the vast majority of polyphagous parasitoids of spruce budworm now appear to be morphologically cryptic host specialists (Smith et al. 2011). While generalists are still present in ecosystems, the trend is for a mass increase in specialization and far less flexibility than previously thought. The visibility of this pattern is driven almost entirely by our inability to identify parasites reliably without molecular tools. An increase in the number of taxa with much more restrictive niches represents a significant challenge for the conservation of biological diversity as they may be much more vulnerable to host (niche) loss.

### Increased flexibility of insectivores

In contrast to the implications for decreased flexibility observed in parasites, molecular methods applied to larger animals frequently show the opposite trend: more flexibility that previously thought and an increasing ‘fuzziness’ in our categorization of ecosystems by trophic levels and feeding guilds. Insectivores have been a model system for the application of high-throughput sequencing of diet, primarily because of the extensive reference database available for terrestrial insects at standardized loci (e.g. cytochrome oxidase *c* subunit 1 – discussed below), making them an obvious and relatively simple target for analysis. In almost all cases, molecular analysis has yielded far more prey groups than previously recognized and far more rare dietary items. For example, half of the families of insects detected in the diet of the Eastern Red Bat were new dietary records but were also detected at very low levels (which, along with morphological crypsis, is a likely reason they were previously overlooked; Clare et al. 2009). These analyses are also providing substantially new insights into habitat use and local adaptations. Environmental indicator species consumed by little brown bats have been detected in guano collected under roosts and used to assess the level of organic pollution and acidification of foraging areas and the type of aquatic system being exploited (Clare et al. 2011, 2014a). This provides an extremely non-invasive method to measure habitat use and quality. Subtle methods of resource partitioning have also been recognized; among *Plecotus* in the UK, seasonal partitioning may be linked to resource limitation (Razgour et al. 2011), *Myotis* in central Europe may partition by physiological difference and prey life stage (Krüger et al. 2014) and an ensemble of bats in Jamaica may use a combination of morphological, acoustic and temporal partitioning of their environment to access resources (Emrich et al. 2014).

Cases of extreme flexibility have also emerged. Endaemic skinks and invasive shrews on Ile aux Aigrettes alternate between mutual predation and resource competition. An intensive investigation showed significant resource overlap among some common prey types raising important questions regarding conservation priorities, habitat use and methods of invasive species control (Brown et al. 2014b). Perhaps, the most extreme case of flexibility investigated thus far is the case of *Glossophaga soricina*, the common tropical nectar bat, which has long been known to occasionally consume insects. Using echolocation to detect and approach a stationary flower, which advertises its presence, is a fundamentally different behavioural task than detecting and tracking flying insects that actively try to avoid capture. However, molecular analysis of insect DNA in the faeces of *G. soricina* indicated they were efficient insectivores consuming many insect species with ears that enable them to detect bat calls (Clare et al. 2013a). The solution to this apparent paradox was that the low intensity echolocation used to locate flowers made them functionally undetectable to insects and thus provided them with a form of stealth echolocation and a predatory advantage (Clare et al. 2013a) and the ability to achieve trophic niche switching. We do not yet know under what circumstances they employ this switch, but it may be determined by relative resource availability and competitive interactions (Tschapka 2004) or be nutrient driven (Ganzhorn et al. 2009), either of which may have significant conservation implications as global change causes species’ ranges and resources to shift and such flexibility decreases species’ sensitivity to such dynamics.

### Fundamentally new insights into network dynamics

These observations do have significant implications for our understanding of food web structure. In the case of the parasites of spruce budworm, a quantified food web demonstrated that overall diversity increased and the level of connectance was reduced when full taxonomic resolution was achieved using molecular approaches (Smith et al. 2011). Connectance describes the degree to which trophic levels are associated; thus, it is unsurprising that this inverse relationship exists. What is more surprising is how important the molecular method may be to our overall conclusions about network dynamics. Network structure is the basis for our understanding of how ecosystems function. However, a recent study concludes that there may be more structural difference due to method than biology. When comparing a parasite network based on traditional rearing methods to one which included molecular documentation of interactions not observed in the laboratory, Wirta et al. (2014) found a threefold increase in the number of interaction types and molecular data significantly altered their conclusions about parasite specificity, parasite load of hosts and the role of predators. Most startlingly, their high arctic rearing web and the molecular web they made for the same system had a fivefold greater level of variation in estimates of vulnerability, a fourfold greater level of variation in linkage density and twice as much variation in generality than the traditional rearing web did when compared to similar networks from around the world including tropical locations. All three measures estimate important network dynamics. Considering just linkage density (the average number of interactions per species), their web generated a higher value than any of the other webs assessed. The fact that even in a species-poor high arctic web, simply adding the missing components detected by molecular means yielded fundamentally new conclusions has vast implications for global assessments of ecosystem dynamics and how resilient or vulnerable they may be to disruption. This is particularly important in conservation biology as we evaluate vulnerable species and ecosystems and prioritize areas for protection and intervention.

## Methodologies, observations and conclusions: how far do we go with the data?

While the techniques are promising and new patterns are emerging, what considerations are there in interpreting such high-resolution data?

### Picking primers and identifying amplicons: ideal target regions

Molecular analyses of species interactions using metabarcoding rely on the amplification of a specific region of interest from unknown mixed taxa. These unknowns are then identified as far as possible. This same principle is used whether the analysis is based on amplifications looking for a specific target (e.g. detecting a particular pest species in a diet) or NGS to assess complete diversity. The process requires that we use primers that are appropriate to our task and that we have some sort of reference or analytical option for the data. Ideally, we would have extremely general primers capable of generating amplicons for all potential species and a curated reference library from which to extract identifications for the sequences; however, this is rarely practical or even possible. It is particularly difficult when trying to assess a completely unknown sample such as we might obtain from a generalist.

The most common approach is to use the most general primers available and then rely on existing databases to act as reference libraries and hope that they were assembled with some taxonomic rigour. GenBank is arguably the largest such database but what it boasts in taxonomic breadth it lacks in taxonomic curation and its ability to identify sequences in volume is severely limited. An alternative is to use smaller more targeted databases and one of the many bioinformatics options for sequence matching. For example, for bacterial and fungal sequencing, most researchers amplify the small-subunit ribosomal RNA V6 hypervariable region or the internal transcribed spacer (ITS-2), respectively (e.g. for a reviews of best practices for fungal community analysis see Huber et al. 2007; Lindahl et al. 2013) and compare these to reference collections, for example SILVA (www.arb-silva.de/) for V6 identification and UNITE (unite.ut.ee/index.php) for ITS identification.

An alternative method is not to identify sequences at all but simply collapse reads into MOTU: molecular operational taxonomic units (Floyd et al. 2002). While this does not help identify the taxa, it does present a method of dealing with both known and unknowns at the same time and is arguably more statistically sound. There are an abundance of MOTU generating methods all with advantages and disadvantages and almost no rigorous testing of their relative performance. Clearly, this is an area in need of substantial exploration.

For animal studies (the focus here), there are other choices for target regions but fewer curated databases. The emergence of DNA barcoding (I restrict this to COI as per Hebert et al. (2003)) in 2003 has led to a decade long campaign to create a highly curated reference library, the barcode of life data systems BOLD (www.barcodinglife.org; Ratnasingham and Hebert 2007) as the store house for these data. While not yet amenable to NGS data, it remains the single largest collection of semicurated homologous DNA regions in existence, comprising approximately 3.4 M sequence reads from 214 K species (at the time of this composition) and has global coverage for some taxa. Thus COI is a common and convenient region to target in these analyses. Furthermore, BOLD hosts a primer registry with more than a thousand primers for the region which can be exploited for adaptations to NGS rather than *de novo* creation. While COI meets the requirement for providing taxonomic resolution, many systems are so overwhelmingly diverse that the number of potential primers required (see the section on bias) makes this a theoretical target but not a particularly practical one without *a priori* hypothesis about composition. Thus, while COI is perhaps the best target for terrestrial macroscopic life and some freshwater applications, marine and parasitic systems remain far too complex for this approach.

Among marine systems, target regions such as ribosomal DNA (12S, 16S, 18S, 28S) are relatively conserved so a single set of primers can amplify a very broad range of phyla (e.g. see Deagle et al. 2007, 2009, 2010, 2013). For example, in the analysis of marine prey in macaroni penguins (Deagle et al. 2007), a combination of 16S, 18S and 28S targets were used which allowed the authors to detect euphausiids, fish, amphipods and cephalopods in the diet of these sea birds during chick rearing. As this is a very broad potential taxonomic assemblage to cope with, a multiregion conserved primer approach is key, but within that diet, there is taxonomic ambiguity because these regions are not efficient at species resolution.

For gastropods, 16S has been used extensively (e.g. Boyer et al. 2013) and for parasites, ribosomal DNA in general may be more applicable (Floyd et al. 2002). In highly complicated systems, a hierarchical approach may be needed (Moszczynska et al. 2009) where a broad target region is initially used to provide a first pass identification and then, based on the outcome, subsequent regions and primer sets can be selected to refine the taxonomic identifications. This approach may also be the best method for environmental assessment where the potential diversity is beyond that of even generalists and all domains of life may be of equal interest.

For herbivores, the problem is doubly complex. DNA barcoding of plants cannot be accomplished using a single region in most cases. Thus, there are at least four common target regions for plant DNA recommended in different combinations (Rubinoff 2006; Chase et al. 2007; Fazekas et al. 2008; CBOL Plant Working Group 2009). While networks for herbivores are being created and this effort is expanding (Soininen et al. 2009; Valentini et al. 2009; Newmaster et al. 2013), the field has been slower to gain widespread use. A combination of the P6 loop of the chloroplast *trnL* region plus ITS was used in conjunction with other biomonitoring approaches to assess the diet of woodland caribou and detect a mixture of lichens, trees, mosses, herbs and grasses (Newmaster et al. 2013). A similar approach in the tropics used *trnL* with ITS1 to confirm the diet of Tapirs (Hibert et al. 2013) and *trnL* to examine the dynamics of prey choice among sub-arctic voles (Soininen et al. 2009).

The trade-off in the reliance on more conserved regions (e.g. ribosomal DNA) is that while it maximizes potential taxonomic coverage, it loses species-level resolution. Another problem with ribosomal regions and some plant regions is that they include introns. NGS platforms are thought to have a high error rate compared with traditional Sanger sequencing and while we can correct for this in coding regions, particularly those that lack introns (e.g. COI), single nucleotide polymorphisms and indels caused through sequencing error in ribosomal genes are extremely hard to detect. The net result is a higher probability of error in defining molecular operational taxonomic units and making taxonomic assignments for these unknowns, decreasing the value of the data for any real biological application. It is possible to use these data effectively, but it requires a higher degree of computational skill and extensive knowledge of the region one is working with.

The net result is that no target is perfect. While COI is ideal for land animals and has all the gold standard requirements for NGS, the primer issues may make it hard to apply in marine systems and parasites. Regions that work well for these systems suffer from a lack of curated databases and the persistence of indels, length variation, etc., making the analysis more complex. At the very least, when picking targets, we must be wary of the limitations. In all cases, there have been far too few studies on how to extract taxonomic information. In some cases, this may have probably led to excessive conservatism (e.g. Bohmann et al. 2011; Clare et al. 2011; Razgour et al. 2011), but the risk of overextending our observations cannot be overlooked (see below).

### Picking primers and amplicons: the long and the short of it and relative biases

There is a trade-off between sequencing a large region to maximize the taxonomic information extracted, and the amount of degradation and contamination in the sample that limits the length that can be recovered. In addition, there is no such thing as a universal primer and those with broad taxonomic applicably are nearly always tested on pure extracts rather than mixtures (e.g. Meusnier et al. 2008; Zeale et al. 2011). While this approach is reasonable for primer development, amplification ability on isolated samples does not predict their behaviour in mixed samples. Target sequence size was also initially constrained by the available NGS platforms themselves. Many did (and some do) only provide very small reads <100 bp in length (Glenn 2011). After the addition of adaptors required by the sequencer, primers to target your region and MID tags which separate samples, frequently 120–140 bp of sequence have already been consumed. This problem has now largely disappeared with most major platforms (Roche Life Sciences, Branford, CT, USA; Illumina, San Diego, CA, USA; Pacific Biosciences, Menlo Park, CA, USA; Life Technologies, Paisley, UK) allowing the production of longer and longer sequences, significantly increasing the options for primers. The optimal target length varies by gene region and taxonomic objective, for example for COI, there is a theoretical lower limit of 109 bp for taxonomic discrimination (Hajibabaei et al. 2006, 2007). However, this assumes a limited taxonomic target and high-quality sequencing reads with few errors; thus, at least for this region, aiming for longer is better. The commonly used Zeale region (Zeale et al. 2011) is 157 bp in length and has been reliable, although appears to have a amplification bias (Clarke et al. 2014, E.L. Clare, personal observation).

When degradation is expected (Deagle et al. 2006), there may be a significant bias for detecting undegraded DNA, which would limit taxonomic recovery, and it is unknown whether degradation would be taxon specific (to both predator and prey) or somewhat random. As such, short amplicons might overcome problems of low amplification success and high contamination by non-prey DNA (Clare et al. 2011) but may be limited in the information they contain and biased towards overestimation of diversity. The source material may thus dictate the choice of primer length, a trade-off between length of amplicon for identification and the impact of DNA degradation.

Perhaps, the ideal solution for both primer bias and primer length is to use a variety of primers yielding a series of lengths in separate PCRs (not multiplexed): short primers to maximize diversity, long regions to maximize and quality check taxonomic identity, different combinations to exploit different biases and, importantly, the ability to estimate the *relative* effects of each in a mixed unknown template. Multiplexing should be avoided so that each reaction has an independent opportunity to occur without interference.

### Volume abundance and biomass

The ultimate methodological achievement in this field will be to generate an accurate and repeatable measure of abundance or biomass within a sample. This is particularly important in conservation biology when we wish to know not only that an interaction occurred, for example did the shrew eat beetle species A, but how often and in what quantities relative to other prey. There are two main methods that have been applied to this problem. Various attempts have been made to use traditional quantitative genetics techniques (qPCR/rtPCR), but these have been problematic (e.g. McCracken et al. 2012), and, while some limited success has been achieved by the very simplest of systems (e.g. Bowles et al. 2011), these cases generally involve extremely limited taxonomic diversity (in this case only four prey), making broader application impractical at this stage.

There have also been attempts to use the number of sequences recovered as a proxy for abundance, for example if the shrew ate more beetles than flies, there should be more beetle DNA in their gut, and thus, more beetle sequences are recovered. There is some evidence for general correlations, but actual evaluations of this method have been unsuccessful (Pompanon et al. 2012; Deagle et al. 2013; Piñol et al. 2014). Even in a system with only three prey fed artificially, apparent differential digestion makes predictions unreliable (e.g. Deagle et al. 2010). While intuitively sequence number should be related to initial biomass, and in some cases is similar, a confusing array of factors come into play which are specific to both the prey and predator, the combination of prey in the diet and the technological steps taken during analysis.

Consider the simple system where a shrew consumes a beetle and a fly in quick succession, there is no DNA from previous prey, bacteria or parasites in the gut and that we are targeting mtDNA. The beetle is much larger than the fly, so we might predict it provides more DNA (beetle > fly); however, the beetle is trapped inside a much harder carapace and so the DNA is harder to extract (fly > beetle). However, the fly, being soft, might be more digested and thus provide less intact DNA (beetle > fly), but the fly degradation might free up more DNA for extraction and PCR (fly > beetle) and so on. Already there are potentially four competing sources of bias, which may influence the amount of DNA. Now consider that fecundity can alter mtDNA content (e.g. a single developing oocyte may increase mitochondrial copy number 1000×, Cotterill et al. 2013), that there are tissue-specific differences in mtDNA (e.g. differential age related copy number variation, Barazzoni et al. 2000) that may or may not survive digestion, that endogenous parasites and bacteria may attack different tissues with different degrees of success if it is protected in an exoskeleton versus soft tissue, and the number of biases exceeds even our ability to predict relative amounts of DNA. In the laboratory, primer biases, targeted sequence lengths, extraction and PCR inhibitors and interactions between DNA in the gut further complicate the chemistry. Analytically Deagle et al. (2013) point out that even the choice of MID code used to separate samples, direction of sequencing and quality filtering have distinct, unpredictable and inconsistent impacts on the recovery of sequences and these interact with each other. In the best of cases, an insectivore might have access to thousands of potential prey and accounting or controlling for this number of variables is inconceivable.

While there does appear to be some correlation between some types of analysis (Razgour et al. 2011), they are too inconsistent to provide reliable suggestions that we can quantify within individual samples and conservation practices should not be set based on this approach given the current risks. It may be possible to assess the relative importance of a single species using targeted amplifications, but the data are still emerging. Molecular analyses, as done now, cannot estimate abundance, biomass or volume within a sample and best practice suggests rare and common items must both be treated as ‘present’. While we cannot estimate sample-based abundance using present methods, we can measure species richness within a sample and frequency across samples. Until we make substantial technological advances, these semiquantitative analyses may be the only way forward in the short term but in themselves have significant limitations (discussed next).

### Overestimation of rare species? The risks of under and over detection of ecological phenomena

Perhaps, the most significant issues to consider before applying such techniques to conservation biology are the potential biases within the data themselves. In particular, there may be a significant overemphasis of rare species using the semiquantitative method (above) and this has a knock-on effect of leading us to over- and underdetect certain ecological phenomena. When interpreting these data, we must be mindful of these effects.

One of the significant advantages of applying molecular analysis to interaction networks has been the ability to detect rare species and thus rare interactions. The resolution is much higher using molecular analyses compared with traditional methods, and because the process can be largely automated, we can accumulate much more information from the same samples with less effort. For example, in our first analysis of bat diet (Clare et al. 2009), we recovered evidence of more than twice the number of families previously known and all new families were the rarest representatives by number of recorded species. Similarly, molecular analysis makes it possible to simultaneously unravel interactions and cryptic complexes (Smith et al. 2006) providing substantial insights into the status of species, which is a vital component of conservation.

While the discovery of new and cryptic relationships is important to the establishment of general trends (see above), there are actual problems with increased resolution. The shift from traditional methods (normally based on morphological analysis) to molecular approaches is argued to be an advantage because most traditional analyses are very limited in their taxonomic resolution (except, for example, those based on culled remains). We may know that predator A ate a fish, but not which fish, and thus, we cannot assess whether the loss of any particular fish species will have an effect on the predator's population status. It is important to realize, however, that, while morphological analyses are limited in their ability to recognize subtle differences and then bias some analyses (e.g. the overdetection of resource overlap), molecular data, which identify prey at the species level, are likely to be biased in the opposite direction (e.g. resource partitioning). As our ability to quantify molecular methods is limited, we will tend to over-represent rare items and underestimate the importance of common items.

Consider two hypothetical species foraging in a single location; they are bound to both encounter a number of common prey and a number of rare prey such that they share common prey but not the rare items. This is not a case of deliberate resource partitioning but encounter stochasticity. If analyses are limited at the level of ‘caterpillar’ or ‘beetle’, it is likely that both species consumed caterpillars and beetles and we conclude little resource partitioning. However, if we boost resolution to ‘species A, species B, species C’ etc. there is a much higher chance that they encountered different species and, as such, it is almost certain that two dietary analyses will contain species that are different. This effect may lead us to conclude that resource partitioning is ongoing in our system. Now add to this problem that within samples, we are limited to presence/absence records and it will quickly become apparent that the effect is greatly amplified because rare and common items are both recorded as ‘present’ and thus given equal weighting. When incorporated into many ecological modelling programs (which expect full abundance measures rather than semiquantitative estimates), which use simulations to determine if overlap is greater or less than expected by chance, measures of resource overlap are likely under-representations of what is ‘real’ while measures of resource partitioning are likely prone to over detection (by the same logic, traditional more restricted ID methods may be biased towards the detection of resource sharing). As such, we must treat minor species-level differences conservatively.

The problem is that some predators probably really are making decisions at the species level and may very well partition on this basis, but not all can do this and not all the time. It is much more likely that predators make adaptive and dynamic decisions at a variety of spatial, resource-driven and taxonomic levels, which are much more complex. So, while traditional approaches are probably underestimating resource partitioning, and molecular approaches probably underestimate resource sharing, knowing how to compensate is not clear. To differentiate these random differences from biologically meaningful partitioning, we must consider at what level an animal perceives its environment. In the case of bats, echolocation likely provides information allowing individuals to perceive insects by size, shape, speed and acoustic reflectivity; it is unlikely that they differentiate subtle morphological differences between species (e.g. see Brigham and Saunders 1990; Barclay and Brigham 1994); however, some specific adaptations (e.g. stealth echolocation) may give certain species special access to some niches (Goerlitz et al. 2010; Clare et al. 2013a); thus, perception is tied to resource use very strongly.

There is considerable debate about the role of rare species in maintaining ecosystem function and while these differences may be important in terms of demonstrating the capacity for behavioural flexibility or stabilizing ecosystem functioning, they may not be important in terms of energetics when these are consumed in low frequency. This is directly related to conservation biology in two specific areas. Rare items and interactions are key components in the debate over the value of biodiversity and are a key measure of ecosystem complexity. However, in applied conservation, where we are interested in the resources required for the persistence of a target species, rare items may contribute little to the diet and are thus less relevant in species’ assessments.

The key then is to recognize the advantage of species-level resolution, while keeping in mind that rare items may be of no biological relevance and selectively neutral for studies of partitioning, yet are biasing ecological models towards the detection of those same effects. Indeed, this is compounded when we analyse only a small subset of the population over a limited timescale. A large sample size may control for overrepresentation of rare prey to some degree (or underrepresentation of common prey as the case may be). As sample size grows, frequency estimates will approach biological reality, although it will remain a problem in cases where taxa are extremely abundant but species poor (e.g. mass-emerging prey such as mayflies). However, a much simpler control may be to remove rare items from statistical analysis altogether and concentrate only on partitioning among common items. This was the approach taken when comparing resource use by endaemic skinks threatened by invasive shrews (Brown et al. 2014b) and, as expected, removing rare items increased estimates of resource overlap. Of course, rare prey may themselves be key in species conservation. Common items in a diet are probably common in the environment, but if rare items confer some specific nutritional component, their loss may be critical. At the very least, these biases are real, present a potentially serious confounding variable and must be considered before drawing conclusions, particularly in regard to management decisions about competition and our assessment of the vulnerability of a species to niche loss.

### A new tool for conservation biology?

With all these potential caveats, the application of molecular tools may seem daunting. From a purely practical point of view, in many cases, molecular methods can be applied extremely non-invasively; for example, to scats found during surveys. This is a significant advantage over regurgitates or direct observation and tracking and can thus be applied to some of the most vulnerable species. There are a variety of cases where these methods are already providing extensive conservation insights.

1. *Vulnerability Assessments*: Niche competition and environmental change are frequently cited as significant factors in the case of population decline. To assess whether a species is vulnerable, accurate niche documentation is required. In particular, understanding how flexible species are within their ecosystems will be a key determinant in establishing their vulnerability to change. For example, understanding network structure in high risk areas will permit us to predict their responses to short (e.g. El Niño events, seasonal changes) and long-term disruptions (e.g. deforestation and climate change), and by doing so, we can accurately assess relative vulnerability of individual species and networks as a whole.

**Figure 3:**
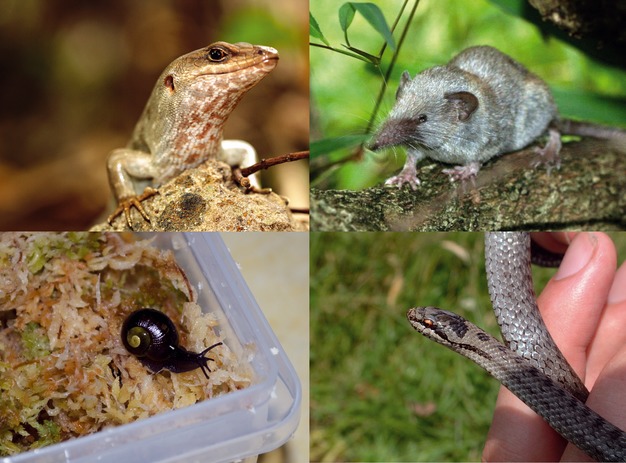
Conservation in action. The application of molecular detection of trophic links is already gaining specific conservation attention. Clockwise from upper left: to look at potential competition for resources between native skinks and invasive shrews on Ile aux Aigrettes, to determine mechanisms of range limitation in smooth snakes and for managed reintroductions of the endangered snail *Powelliphanta augusta*. Photographs used with permission: skink and shrew – Nik Cole – Durrell/MWF, smooth snake – W.O. Symondson, *P. augusta* – Stephane Boyer.

For example, Smooth snakes (*Coronella austriaca*, Fig.3) are widespread in Europe but have a limited distribution in the UK. Recent molecular analyses have suggested a possible reason for this based on resource availability and a developmental dietary shift (Brown et al. 2014a). These analyses suggest that predation on mammals increase as the snakes reach adulthood, but as juveniles, they are more dependent on reptiles. Similar shifts were not seen in the more common sympatric grass snakes. This suggests more resource specificity in the smooth snakes and that their range and density may be limited by reptile densities required to support juveniles and that reptile population variation may have a strong effect on the population dynamics and persistence of *C. austriaca*.

1. *Restoration Ecology*: Species (re)introductions and habitat restoration rests on the assumption that such programmes can provide adequate resource provisions for focal species. An accurate measure of dietary requirements of the focal species via molecular methods can then be used to identify sites, which can provide the appropriate ecological requirements.

For example, in New Zealand, the highly endangered land snail *Powelliphanta augusta*'s natural range is found on Mount Augustus on the western portion of the Stockton Plateau. This area has been heavily disturbed through open-cast coal mining, and there have been numerous court challenges concerning environmental issues in the area. One specific effort to preserve biodiversity is through managed translocations or maintaining captive collections of *P. augusta* (Fig.3). The eventual hope is that this species might be re-introduced following environmental restoration. *Powelliphanta augusta* is thought to consume mostly earthworms but little as known about dietary variability or preferences. Next-generation sequencing efforts have been used to try and determine the precise resource requirements for *P. augusta* to aid in future restoration plans. Snails were briefly taken into captivity (Boyer et al. 2013), and faeces were collected. The authors used targeted 16S metabarcoding and NGS using the Roche 454 system. They determined that most individuals had eaten more than one species of earthworm, but that a few specific worms were common to almost all individuals and are likely important dietary components, but that foraging appears somewhat random. This has relevance immediately in captive feeding programmes meant to temporarily maintain this species but also in longer term plans for reintroduction.

1. *Identification of Key Food Web Links*: Molecular resolution may fundamentally change our assessment of ecosystem network dynamics. In particular, the key dynamic measures (vulnerability, linkage density and generality) describe the degree with which species at higher levels use species at lower levels, the average number of interactions any one species may make and the average number of species at lower trophic levels interacting with higher levels. Areas where these measures include particularly threatened or unusual species may be used to flag particularly vulnerable ecosystems in need of either conservation or direct restoration. Similarly, these links may establish ecosystem pathways correlated with toxicity and contamination within the environment.

For example, tracing environmental contaminates through ecosystems can be extremely difficult. In a recent experiment (Šerić Jelaska et al. 2014), the diet of carabid beetles was determined by PCR-based gut analysis in a forest community using a variety of primer pairs of differing level of target specificity. They revealed a diet of earthworms and slugs as well as smaller invertebrates. Analyses of the beetles and the prey showed metal bioaccumulation (lead, cadmium and mercury), which correlated with seasonal dietary changes. The authors suggest that carabids may be useful bioindicators in contaminated sites and that they and their prey are both in turn consumed by birds and mammals.

1. *Impacts of Invasive Species*: The impact and persistence of invasive species is a key concern in conservation ecology. Because molecular methods can be applied very rapidly, we have the capacity to quickly assess the ecological role of an invasive taxon in its home environment and use this information to evaluate potentially vulnerable species at the site of invasion. This rapid-response model can highlight areas of direct competition to establish which native species might be vulnerable and why to form a more targeted response to invasion.

For example, Telfair's skinks (Fig.3) were once found across Mauritius but now have a very restricted range. They have been introduced from Round Island onto Ile aux Aigrettes to establish a new population making them less vulnerable to random events. Ile aux Aigrettes has been cleared of rats but invaded by Asian Musk Shrews (Fig.3). NGS approaches were recently used to assess potential niche competition and mutual predation between these two (Brown et al. 2014b). While most prey were not shared, niche overlap was significant, suggesting potential for strong competition when food is limited; indicating that the removal of shrews from the island should be a strong priority to encourage the establishment of the skink population.

### Where do we go from here?

There are number of areas that need to be advanced for molecular analysis to achieve its full potential for the study of interacting ecological systems and to be incorporated into conservation and management. Technologically, we need to expand reference libraries and optimize molecular and informatics protocols to maximize taxonomic breadth and coverage using those same libraries. In particular, specific steps in the analytical chain that have been ‘borrowed’ from genome assembly need to be quantified to see whether and how they impact on ecological analyses. For example, it is standard practice to discard all unique haplotypes during quality filtering. This assumes that sequencing depth is sufficient so that any haplotype that is rare is likely a result of sequencing error or an amplification chimera. However, scanning such disregarded sequences clearly demonstrates there are good data in there (E.L. Clare, personal observation) and logic tells us that rare items may generate rare haplotypes. While removal of these is ‘standard practice’, this needs to be evaluated in a quantitative framework to see whether and how it influences eventual ecological analyses; are our assessments made more vulnerable by including bad data or dismissing good data?

Methodologically, we need to scale up. Most analyses are still considering very limited systems; most consider only one taxon and its resource (Newmaster et al. 2006; Deagle et al. 2007; Clare et al. 2014b), a few have considered two interacting predators (Bohmann et al. 2011; Razgour et al. 2011; Brown et al. 2014a,b; Krüger et al. 2014) and rare cases have considered slightly wider ensembles (Emrich et al. 2014; Wirta et al. 2014). None have yet considered an entire community, multiple trophic levels or ecosystem-level complexity. These are now within our technological ability and are certain to provide extremely novel insights into ecosystem-level processes, network dynamics and flexibility. Characterizing an interaction network to this degree is challenging, particularly in tropical systems where taxonomic richness is high and species of interest are diverse, but if behavioural flexibility and network complexity increase stability, these factors may be especially important, particularly in areas at risk from habitat disruption.

**Figure 4:**
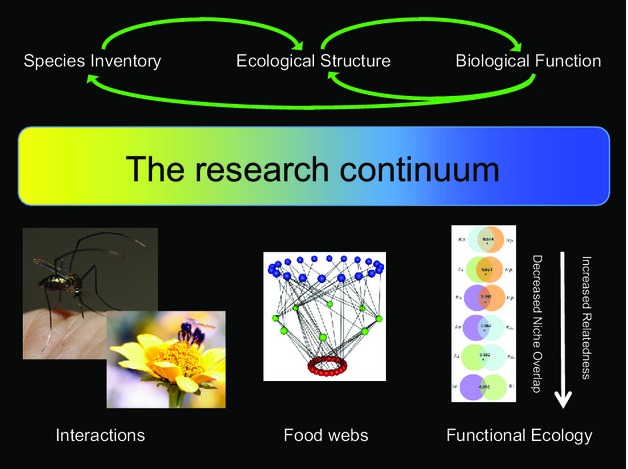
The accumulation of molecular data to assess species inventories has given us the capacity to assemble high-resolution measures of ecological structure such as food webs. From these, we can measure specific aspects of biological function such as specific cases in functional ecology. However, the analytical continuum should also allow us to use functional ecological principles to predict aspects of network structure, expected species interactions and in turn actually predict or identify missing species from inventories. This last point is critical in conservation biology allowing us to identify key factors promoting species vulnerability or persistence (Photographs used with permission: M. Brock Fenton).

Moving towards fully quantified ecological networks will have significant impact on both functional ecology and functional genomics and may increasingly unify these fields. The creation of reference taxonomic databases as part of biodiversity inventories has led directly to our ability to generate high-resolution interaction networks. Networks themselves are an excellent method of elucidating biostructure and can serve a dual role. First, they highlight unusual interactions, for example species that are particularly specialized or have extreme characteristics or groups of species convergently acquiring similar characteristics. This feature makes them a perfect way of locating interesting evolutionary patterns and species with particular genomic features associated with trophic roles. Second, well-parameterized networks provide direct information on ecosystem stability, flexibility and capacity for adaptation under environmental change. This component makes them vital for our understanding of functional ecology and conservation biology. Finally, these factors form a natural feedback – better understanding of functional genomics and ecology will predict ecosystem structure and thus biodiversity inventory (Fig.4).

## Conclusions

Molecular methods, particularly high-throughput sequencing, present challenges, but the insights gained from these tools are generating fundamentally new observations and conclusions about the dynamics of ecosystem structure. Cases where these methods have been applied are rare, but the capacity to provide fast and reliable biomonitoring tools suggests that these technologies will be extremely useful in multiple areas of conservation biology. Careful application will be key to ensuring successful outcomes.
